# Transperitoneal laparoscopic approach for retrocaval ureter

**DOI:** 10.4103/0972-9941.26647

**Published:** 2006-06

**Authors:** H K Nagraj, T A Kishore, S Nagalakshmi

**Affiliations:** Department of Urology, M. S. Ramaiah Medical College, Bangalore - 560 054, India

**Keywords:** Hydronephrosis, laparoscopy, pyeloplasty, retrocaval ureter, retroperitoneoscopy, ureteroureterostomy

## Abstract

We had a 14 year old boy, who presented with recurrent attacks of right loin pain. Investigations revealed a retrocaval ureter. A transperitoneal three port laparoscopic approach was undertaken. The retrocaval portion of ureter was excised. A double J stent was placed laparoscopically and ureteroureterostomy was done with intracorporeal suturing. The patient was discharged after 72 hours and the stent was removed on the 15^th^ day. Follow up showed regression of hydronephrosis. We recommend this approach compared to open surgery, as it offers several advantages compared to conventional open surgery like decreased postoperative pain, decreased hospital stay and a cosmetically more acceptable surgical scar.

## INTRODUCTION

Retrocaval ureter is an uncommon venous anomaly, in which the right ureter courses posterior to the inferior vena cava (IVC) and partially encircles it. It results from persistence of the posterior cardinal venous system that anomalously forms the IVC and subsequently courses anterior to the ureter for a variable distance. This can cause varying degrees of ureteral obstruction and surgical intervention is often necessary.

## CASE REPORT

**Figure 1:**
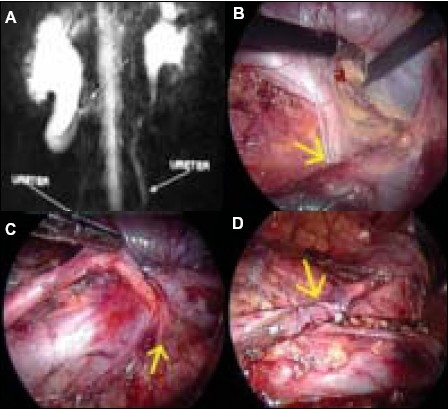
A. Showing MRI of retrocaval ureter showing “fish hook” deformity, B. Dilated proximal ureter dissected out upto the lateral border of IVC. C. Distal ureter a mobilized in the interaortocaval region. D. Ureteroureterostomy done with 4.0 vicryl by intracorporeal suturing.

A 14-year-old boy presented with recurrent attacks of loin pain of two years duration. Ultrasound showed right hydronephrosis and dilatation of proximal ureter. MR Urogram showed classical seahorse appearance [Figure 1A]. Patient was placed under general anesthesia. A Foley catheter No. 12 Fr size was inserted. The patient was placed in the left lateral position. A three port approach with primary port at the umbilicus, one 5 mm port midway between the umbilicus and the medial costal margin and a 5 mm. port midway between the anterosuperior iliac spine and the umbilicus, was used. The line of Toldt was incised and the ascending colon was reflected medially. After exposing the retroperitoneum, the ureter was identified coursing posterior to the inferior vena cava. The dilated ureter was identified and dissected out upto the lateral border of IVC. [Figure 1B]. The lower ureter also was mobilized in the interaortocaval region [Figure 1C]. The retrocaval portion was dissected out and excised. The distal ureter was spatulated. A stay suture was taken from the proximal ureter, which stabilized the ureter. A double J stent was inserted in an antegrade manner laparoscopically. Then a ureteroureterostomy was done with 4.0 vicryl by intracorporeal suturing [Figure 1D]. A closed suction drain was placed and the operative site was retroperitonealized. The duration of the surgery was 100 minutes. The drain was removed after 48 hours. The patient was discharged after 72 hours. Stent removal was done on the 15^th^ postoperative day and retrograde pyelogram showed normal ureter. Post-operative follow up with ultrasound showed that hydronephrosis had regressed.

## DISCUSSION

The classical treatment for retrocaval ureter consists of separating the ureter, re-anastomosing its stumps and replacing the ureter in its usual position while maintaining its patency. The open ureteroureterostomy remained the gold standard surgical approach to treat the retrocaval ureter for many years. Matsuda *et al* first performed the laparoscopic ureteroureterostomy for a retrocaval ureter in 7.5 hours using five laparoscopic ports.[1] Salomon *et al*. in 1999 reported the first case of purely retroperitoneal laparoscopic repair of a retrocaval ureter, which suggested that the retroperitoneal laparoscopy represented the more direct approach to the urinary tract.[2] The advantages of this approach as proposed by the authors was direct approach to urinary tract, decreased operative time and lack of hindrance from other intra-abdominal organs. Gupta *et al* employed a three-port retroperitoneoscopic and found this approach to be safer, easier and less time-consuming and that it provided direct access to the ureter and IVC, while avoiding spillage into the peritoneal cavity.[3] The shortest operative time was reported by Gupta (3.5 hours), the other cases took 3.75–9.3 hours. More recently, Tobias-Machado *et al*. reported a case of retroperitoneoscopic surgery coupled with extracorporeal uretero-ureteral anastomosis.[4]

The main limiting factor for both the transabdominal and the retroperitoneal laparoscopic repair of the retrocaval ureter, was the intracorporeal anastomosis of the ureter that significantly increased the surgical time. With increasing experience in intracorporeal suturing, operative time can be minimized. However, our report demonstrates that laparoscopic ureteroureterostomy may be safely and effectively performed in a reasonable operative time, with return to normal activity within one week postoperatively. The choice of surgical approach also depends upon the surgeon's experience and preferences.

Comparisons between historical reports about open surgery and laparoscopic surgery for retrocaval ureter have clearly shown the advantages of minimally invasive approaches like less intraoperative bleeding, a shorter post-operative hospital stay, reduced postoperative pain, earlier return to daily activities and a significant superior aesthetic effect, while preserving therapeutic efficacy [Table 1]. Due to paucity of cases reported worldwide, there is no sufficient knowledge about the preferable laparoscopic approach to correct this rare disease.

**Table 1:** Previously reported cases of laparoscopic repair of retrocaval ureter

Authors	Approach	Number of ports	Time of surgery
Matsuda *et al*[1]	Transperitoneal	5	7.5 hours
Salomon L[2]	Retroperitoneal	4	4.5 hours
Gupta *et al*[3]	Retroperitoneal	3	3.5 hours
Tobias-Machado M[4]	Retroperitoneal	3	130 minutes
Polascik TJ[5]	Transperitoneal	3	3.5 hours
Nagraj *et al*	Transperitoneal	3	100
